# Dynamics of serum antibodies to and load of porcine circovirus type 2 (PCV2) in pigs in three finishing herds, affected or not by postweaning multisystemic wasting syndrome

**DOI:** 10.1186/1751-0147-52-22

**Published:** 2010-03-19

**Authors:** Inger M Brunborg, Caroline Fossum, Bjørn Lium, Gunilla Blomqvist, Elodie Merlot, Anne Jørgensen, Lena Eliasson-Selling, Espen Rimstad, Christine M Jonassen, Per Wallgren

**Affiliations:** 1 National Veterinary Institute, PO Box 750 Sentrum, N-0106 Oslo, Norway; 2 Section of Immunology, BVF, Swedish University of Agricultural Sciences (SLU), PO Box 588, SE-751 23 Uppsala, Sweden; 3 National Veterinary Institute, SVA, SE-751 89 Uppsala, Sweden; 4 INRA, UMR1079, F-35000 Rennes, France; 5 Norwegian Pig Health Service, PO Box 396 Økern, N-0513 Oslo, Norway; 6 Swedish Animal Health Service, Kungsängens gård hus 6B, 753 23 Uppsala, Sweden; 7 Norwegian School of Veterinary Science, PO Box 8146 Dep, N-0033 Oslo, Norway; 8 Department of Clinical Sciences, Swedish University of Agricultural Sciences (SLU), PO Box 7070, SE-750 07 Uppsala, Sweden

## Abstract

**Background:**

Despite that PMWS commonly affects pigs aged eight to sixteen weeks; most studies of PMWS have been conducted during the period before transfer to finishing herds. This study focused on PCV2 load and antibody dynamics in finishing herds with different PMWS status.

**Methods:**

Sequentially collected blood samples from 40 pigs in each of two Swedish (A and B) and one Norwegian (C) finishing herds were analysed for serum PCV2-load and -antibodies and saliva cortisol. The two Swedish herds differed in PMWS status, despite receiving animals from the same sow pool (multi-site production). However, the PMWS-deemed herd (A) had previously also received pigs from the spot market. ResultsThe initial serum PCV2 load was similar in the two Swedish herds. In herd A, it peaked after two weeks in the finishing herd and a high number of the pigs had serum PCV2 levels above 10^7 ^per ml. The antibody titres increased continually with exception for the pigs that developed PMWS, that had initially low and then declining antibody levels. Pigs in the healthy herd B also expressed high titres of antibodies to PCV2 on arrival but remained at that level throughout the study whereas the viral load steadily decreased. No PCV2 antibodies and only low amounts of PCV2 DNA were detected in serum collected during the first five weeks in the PMWS-free herd C. Thereafter a peak in serum PCV2 load accompanied by an antibody response was recorded. PCV2 from the two Swedish herds grouped into genotype PCV2b whereas the Norwegian isolate grouped into PCV2a. Cortisol levels were lower in herd C than in herds A and B.

**Conclusions:**

The most obvious difference between the Swedish finishing herds and the Norwegian herd was the time of infection with PCV2 in relation to the time of allocation, as well as the genotype of PCV2. Clinical PMWS was preceded by low levels of serum antibodies and a high load of PCV2 but did not develop in all such animals. It is notable that herd A became affected by PMWS after errors in management routine, emphasising the importance of proper hygiene and general disease-preventing measures.

## Background

A role of porcine circovirus type 2 (PCV2) in the etiology of postweaning multisystemic wasting syndrome (PMWS) was first observed in Canada in 1991, and described in the late 1990s [1]. Since then, PMWS has been diagnosed globally [2], but no single factor that triggers PMWS in PCV2-infected pigs has been identified. Attempts to relate the occurrence of PMWS to infection with PCV2 of a certain genotype have not been conclusive and the spread of PMWS is still enigmatic [3]. PCV2 seems to be ubiquitous in pigs [2], and the ambiguity of PMWS is evident in multi-site sow pool systems which can include both healthy and PMWS-affected satellites, despite that the sows are mixed at a common sow hold during the dry period, and alter between farrowing sites [4].

PMWS appeared comparatively late at the Scandinavian Peninsula and was not diagnosed in Sweden or Norway until 2003 when two Norwegian herds were affected by PMWS [5]. These herds were stamped out during the spring/summer of 2004, and until February 2008 no new case of PMWS was diagnosed in Norway as also demonstrated by screening programs performing necropsies on runt pigs [6]. In Sweden, PMWS was diagnosed for the first time in December 2003 [7]. Three years later, 124 herds had been diagnosed with PMWS and the disease was regarded as endemic in the country [8]. Thus, the spread of PMWS was interrupted in Norway but prevailed in Sweden, and in 2007, when the present study was conducted, PCV2 was present in pigs from both countries but PMWS was only diagnosed in Swedish herds.

Pigs can be affected by PMWS up to 16 weeks of age [2,9,10], which includes at least the first month in the finishing unit. As the mean economical loss for each dead finishing pig exceeds that of a dead weaner by 50% [11], and because the mortality figures due to PMWS in Sweden have been fairly equal in all categories of herds [8], the economic impact of PMWS is likely to be higher in finishing herds than in piglet producing herds. Despite this, most studies of PMWS have focused on the period from weaning until transfer to finishing herds. In a recent field study conducted in Denmark and Spain it was shown that the majority of cases with PMWS in Denmark occurred in the nurseries whereas the incidence of PMWS in Spain was highest in the finishing facilities [12].

The primary objective of the present field study was to investigate the relation between PCV2 load and levels of antibodies to the virus in serum collected from finishing pigs housed in herds with and without PMWS. As stress level has been suggested to contribute to the developments of PMWS [13], saliva was collected for the assessment of cortisol levels. Two Swedish herds, one affected with PMWS (A) and one not affected (B), were investigated. These herds had equally sized finishing units and recruited growers from different herds within the same Swedish sow pool (a multisite production system where piglet producing herds lease pregnant sows from a shared central unit). For comparison a Norwegian finishing herd (C) recruiting growers from a Norwegian sow pool free from PMWS was included. The study was conducted in 2007 when PMWS was endemic in Sweden, but no clinical case of PMWS was diagnosed in Norway.

## Materials and methods

### General health status and description of herds

Both Sweden and Norway are free from diseases listed by the Office International des Epizooties (OIE), including Aujeszky's disease (AD) and porcine reproductive and respiratory syndrome (PRRS), as well as from porcine endemic diarrhoea (PED) and transmissible gastro-enteritis (TGE).

**Figure 1:**
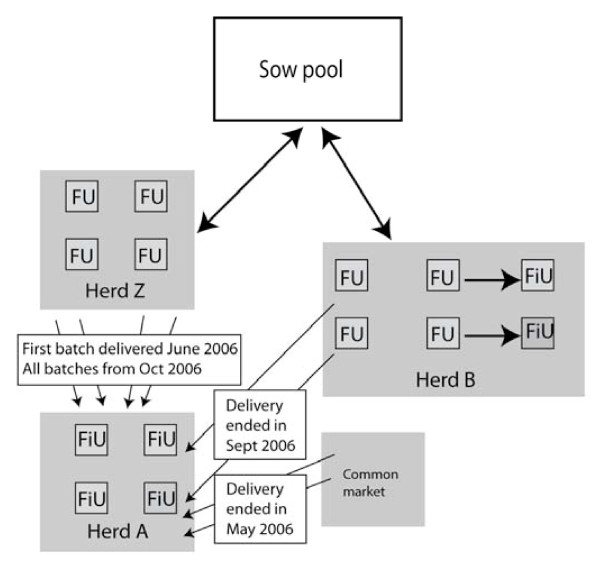
**The supply of growers to herd A**. A change of pig supplier was initiated because Herd Z could supply herd A with all finishing pigs. During this process the empty time between batches was decreased giving little or no time for hygienic measures. Herd A was diagnosed with PMWS in February 2007, while finishing pigs in herd B remained free from PMWS. Herd B and herd Z were neighbours and received pregnant sows from the same sow pool. The distance to herd A was 115 km for both herds. (FU = farrowing units, FiU = finishing units).

The three herds (A, B and C) included in the study, were selected in order to match in size, type and management. The sows were not vaccinated against PCV2, no vaccinations of the grovers were performed. and the feed was free from antibiotics. All three herds effectuated all in-all out production in cycles of 16 weeks in units with 350 to 400 pigs, and recruited growers at the weight of about 30 kg from piglet producing satellite herds in sow pools. The trade with pigs within the Swedish sow pool is illustrated in Figure 1 and a brief description of the herds is given below.

Herd A was a specialised Swedish finishing herd with 4 units, recruiting 400 growers to one of the units every 4^th ^week. The herd used to recruit every second batch of growers from herd B until September 2006, and the batches in-between these from the open market. In order to receive all growers from the same source, herd A contracted herd Z that was a specialised piglet producing satellite within the same sow pool as herd B. The first batch from herd Z arrived in June 2006, and from October 2006 all growers emanated from that herd. Herd A generally cleaned and washed every unit between consecutive batches, but during the process of changing piglet supplier (from herd B and open market to herd Z), occasionally market weight finishing pigs left a unit in the morning and new growers arrived in the afternoon, leaving little or no time for hygienic measures. In accordance with the EU-definition [14], herd A was diagnosed with PMWS in February 2007.

Herd B was an integrated Swedish farrow to finish herd with two finishing units, recruiting 400 growers to one of the units every 8^th ^week. The herd had four farrowing units and farrowing took place every 4^th ^week. At every second farrowing, herd B recruited own pigs to one of the two finishing units that were located less than 100 m from the farrowing units. Herd B cleaned and washed every unit between consecutive batches, and the empty time between batches had been 5.7 ± 0.6 days for the last 15 batches (120 weeks). Herd B was, and by September 2009 still is, free from PMWS.

Herd C was a recently established Norwegian finishing herd with two identical units each with 350 pigs, recruiting pigs to both units every 16^th ^week. The herd recruited growers from piglet producing satellites in a Norwegian sow pool. It cleaned and washed each unit between consecutive batches, and the empty time between the seven first batches was 4.3+1.5 days. By September 2009 this herd is still free from signs of PMWS.

### General study design

This study was approved by the ethical committee in Uppsala, Sweden (License C120/7). The study was carried out during the spring of 2007, one month after herd A had been diagnosed with PMWS. In each herd, 40 pigs in one batch were scrutinised. One week after arrival to the finishing unit, 4 randomly selected pigs from each of 10 pens were given an identity by ear tagging. Blood samples without additive were collected weekly from each of these pigs by jugular vein puncture during weeks 1 to 5 after arrival in all herds, and the serum samples were stored at -20°C until analysed. Two additional samplings were carried out in herd C at weeks 9 and 11 after arrival. Clinical signs of disease were recorded weekly for the 40 pigs. Clinical signs that could indicate PMWS were examined and the pigs were accordingly referred to as "healthy", "thin" (under weight) and/or "hairy" (having a rough appearance). The chest perimeter was measured to estimate the individual growth rate and every pig suspected for PMWS was culled and the clinical diagnosis was either confirmed or rejected by necropsy. To measure chronic stress, saliva samples were collected at week five from ten pigs housed in pens adjacent to the experimental pigs to measure cortisol levels. The saliva samples were collected by letting the pigs chew on cotton swabs (Salivette, Sarstedt AG, Nümbrecht, Germany) until moistened. The cotton buds were kept on ice until centrifuged for 15 minutes at 300 *g*, 4°C, and the recovered liquid was stored at -20°C until analysed. All saliva samples were collected at mid-day to avoid differences due to the normal diurnal variation in cortisol levels.

### Measurement of saliva cortisol levels

The cortisol was measured using a luminescence immunoassay kit (LIA, IBL, D-22335 Hamburg, Germany). The assay sensitivity was 0.15 ng per ml. The inter- and intra-assay coefficients of variation were 7.8% and 6.1%, respectively, at 2.1 ng/ml.

### Nucleotide sequencing of isolates

The virus isolates from the three herds were determined by nucleotide sequencing of the entire genome by two overlapping PCR products. Sequences were acquired from three pigs from each herd and a consensus sequence was created. Primers used for amplification were PCV2-ORF1-1673 towards PCV-F-1319L21, and PCV2-Cap-sense towards PCV-C-1256U21 (Table 1). Briefly, a 50 μl PCR reaction (0.3 mM dNTP, 0.5 μM of each primer, 1.5 U HotStar Taq DNA polymerase in a 1× PCR buffer provided with the kit) (HotStar Taq DNA Polymerase, Qiagen, Germantown, MD, USA) was run with the following program (95°C for 15 min followed by 41 cycles of 94°C for 50 sec, 55°C for 60 sec and 72°C for 45 sec (PCV-C-1256U21) or 95 sec (PCV-F-13119L21), with a final elongation step of 5 min at 72°C). Inner primers used for sequencing are displayed in Table 1. Nucleotide sequencing was run on the Avant 3100 (Applied Biosystems, Foster City, CA, USA) and sequence analysis was performed using Sequencing Analysis 5.2 Patch 2 (Applied Biosystems), Sequencher 4.5 (Gene Codes Corporation, Ann Arbor, MI, USA) and MEGA 3.1 http://www.megasoftware.net. The sequences were compared pair-wise at both the nuclotide and amino acid levels using Lasergen and MegAlign Software, version 1.13 (DNASTAR). Multiple alignments were performed using the CLUSTAL W program.

**Table 1:** Primers used for amplification of PCV2 DNA and nucleotide sequencing.

Primer designation	Primer sequence
PCV-C-1256U21	3'-ATA GCG GGA GTG GTA AGA GAA-5'
PCV-F-1319L21	3'-GCA ACA GCC CTA ACC TAT GAC-5'
PCV2-Cap-sense	5'-ATG ACG TAT CCA AGG AGG CG-5'
PCV2-ORF1-415	3'-CTG TGA GTA CCT TGC TGG AGA-5'
PCV2-ORF1-501	3'-GCT CAC TTT CAA AAG TTC AGC-5'
PCV2-ORF1-804	3'-CTG ATT ACC AGC AAT CAG ACC-3'
PCV2-ORF1-881	3'-CCT CCG ATA GAG AGC TTC TAC-3'
PCV2-ORF1-1673	3'-TGG CCA AGA TGG CTG CGG-5'

### Real-time PCR for quantification of PCV2

DNA was isolated, and a quantitative real-time PCR was run on all serum samples. Briefly, nucleic acids were isolated from 200 μl serum using a NucliSENS^® ^easyMAG™ nucleic acids extractor (bioMérieux, Durham, NC, USA), and eluted in 55 μl elution buffer. Following sequencing of the viruses found in each herd, tailored primers and probe based on a previously described protocol [15], were used for unbiased amplification and absolute quantification of PCV2 DNA. In brief, forward primer PCV-E-1319L21 and reverse primer PCV-A-1256U21 in combination with TaqMan2-PCV2 were used for the Swedish samples (herds A and B). The Norwegian samples (herd C) were analysed using forward primer PCV-D-1319L21, reverse primer PCV2-84-1256U21 and TaqMan-1286-1314 as probe. For each sample, 2.5 μl of the eluate was run in a 25 μl reaction with an annealing step at 60°C, on an MxPro 3005 PCR machine (Stratagene, Agilent Technologies, Inc., Santa Clara, CA, USA). Results are given as number of DNA copies per ml serum.

### Detection of PCV2 specific serum antibodies

Antibodies to PCV2 were measured in individual serum samples using an immunoperoxidase monolayer assay (IPMA) technique previously described [16] with slight modifications [17]. The serum samples were diluted in serial two-fold steps (from 1:10 to 1:20,480) in PBS containing 0.05% Tween and 5% fat-free milk powder. The results are presented as 10 log values of the highest dilution with positive reaction in the IPMA. Titres less than 1/40 (10^1.6^) were considered as negative.

### Statistical analysis

Quantitative real-time PCR-samples below the detection limit of 1.1 × 10^3 ^copies per ml serum were set to 550 (0.55 × 10^3^) copies per ml serum, representing the mean of the values, and likewise, the samples calculated to be between the detection limit and the quantification limit of 1.1 × 10^4 ^copies per ml serum [15], were set to 6.05 × 10^3 ^copies per ml serum. Fisher test was used for comparison of number of animals with viral load above 10^7 ^PCV2 DNA copies per ml serum. To evaluate differences in PCV2 load, levels of antibodies to PCV2, and production data of the pigs in the three herds, groups were compared pair wise using double sided t-tests (two sample tests with unequal variation).

## Results

### General health status and performance

Moderate lameness and coughing were observed in a few pigs in each herd, but the general health status and performance were high in all herds. During the early rearing period, this was demonstrated by steadily increasing chest perimeters of the 40 principals in each herd. From weeks 1 to 5 the chest perimeters increased with 16.1 ± 5.4 cm, 14.3 ± 2.7 cm and 12.7 ± 1.8 cm in herd A, B and C, respectively (A and B vs. C; p < 0.01, A vs. B; p = 0.07).

All three herds had a high daily weight gain and the mean daily weight gain of pigs that reached market weight in herd A was not affected during the period when the herd was diagnosed with PMWS (Table 2). However, the mortality during the rearing period increased from 1.8 ± 0.5% to 2.9 ± 1.3% (p < 0.01), and the prevalence of pigs slaughtered at underweight increased from 1.7 ± 1.0% to 3.6 ± 2.5% (p < 0.05). The mean mortality in herds B and C was less than 1% throughout the study.

**Table 2:** Production data for a Swedish finishing herd (A) during the course of PMWS.

	Herd A				Herd B	Herd C
**Health status regarding PMWS**	***Healthy***	***Suspected***	***Deemed***	***Healthy***	***Healthy***	***Healthy***

Arrival of first and last batch in category	Jan05 -- Oct05	Nov05 -- Oct06	Nov06-Feb08	Mar08 -- Sept08		

Number of batches	11	13	18	6	11	7

***Source of finishing pigs:***						

Herd B (number of batches)	6/11	6/13	0/18	0/6	11^1^/11	Norwegian

Open market (number of batches)	5/11	4/13	0/18	0/6	0/11	sow

Herd Z (number of batches)	0/11	3/13	18/18	6/6	0^1^/11	pool

Batches not preceded by empty days (n)	3	5	4	0	0	0

Mean empty time between batches (days)	4.0 ± 2.8	3.8 ± 3.6	4.4 ± 2-9	5.8 ± 1.2	5.7 ± 0.6	4.3 ± 1.5

Pigs/batch (n)	385 ± 1	385 ± 1	385 ± 1	385 ± 1	389 ± 17	704.7 ± 4.3

Arrival weight (kg)	32.0 ± 3.4^a ^**	31.9 ± 4.0^a ^**	28.0 ± 3.6^b^	27.4 ± 2.5^b^	31.1 ± 1.8	29.0 ± 0.9

Slaughter weight carcas (kg)	87.9 ± 1.6	87.5 ± 2.3	89.3 ± 2.1	87.4 ± 1.8	86.1 ± 2.1	80.3 ± 2.6

Rearing period (days)	104.8 ± 3.5	102.5 ± 5.4	106.3 ± 4.6	103.8 ± 5.6	104.4 ± 5.8	98.4 ± 4.7

Percentage meat of carcas (%)	57.5 ± 0.8	57.8 ± 0.3	57.8 ± 0.7	57.3 ± 0.4	57.8 ± 0.7	56.3 ± 0.6

Mortality, mean (%)	1.8 ± 0.5^a ^**	2.2 ± 0.9^a ^*	2.9 ± 1.3^b^	2.4 ± 1.0^b^	0.5 ± 0.6	0.6 ± 0.5

Mortaliy, range (%)	1.1 -- 2.6	1.0 -- 3.6	1.0 -- 6.2	0.8 -- 3.4	0.0 -- 2.2	0.1 -- 1.4

Condemned at slaughter (%)	0.5 ± 0.5	0.5 ± 0.6	0.4 ± 0.5	0.6 ± 0-6	0.2 ± 0.2	0.5 ± 0.5

Daily weight gain (g)	910 ± 30	911 ± 35	914 ± 35	898 ± 30	886 ± 21	924.7 ± 32.3

Slaughter weight < 73 kg (%)	1.7 ± 1.0^a ^**	2.4 ± 1.5	3.6 ± 2.5^b^	5.3 ± 3.4	3.5 ± 1.6	No records

Slaughter weight < 73 kg, range	0.5 - 3.5	0.3 -- 4.9	0.1 - 10.3	1.6 -- 9.9	0.5 -- 5.9	No records

Results on the same line with different letters differ significantly from each other; p < 0.05 (*) or p < 0.01(**)

For comparison, corresponding data are given for a herd (B) that received animals from the same sow pool as herd A but remained free from PMWS, and for a healthy Norwegian finishing herd (C). The country of Norway was free from PMWS when the study was conducted. Herd B and herd Z received sows from the same sow pool, *i.e*. from the same source.

An increased frequency of runts, wasting pigs and mortality was observed during the period when herd A changed piglet supplier from herd B and the open market to herd Z during June to October in 2006. Due to a 14 day discrepancy between farrowing periods in these herds, less than 24 hours were allowed between batches at several occasions (Table 2). In February 2007, the mortality in a batch reached 4.3% and herd A was then officially diagnosed with PMWS based on clinical and laboratory findings. At that time pigs in the eldest batch had arrived at the herd in November 2006. However, batches with increased mortality had been observed earlier, peaking at 3.6% in a group that arrived by the end of November 2005. Therefore, batches arriving from that time until the herd was officially diagnosed with PMWS are referred to "suspected" for PMWS in Table 2. Herd A was officially declared free from PMWS at the end of February 2008, and batches arriving from March 2008 are again referred to as healthy (Table 2).

### Clinical signs

One week after arrival, two pigs in herd A expressed clinical signs resembling PMWS (under weighted = "thin" or having a rough appearance = "hairy"). At the following observations such signs were observed in 2-6 pigs. Five percent (2/40) of the pigs in herd A developed clinical PMWS (pig number 13 at day 18, and pig number 6 at day 35). Both pigs expressed an acute wasting that was also mirrored by a reduced chest perimeter (from 67 to 58 cm within 4 days in pig 13, and from 65 to 61 cm in pig 6 during the last week), and enlarged inguinal lymph nodes. Both pigs were euthanized during wasting and PMWS was confirmed by necropsy by fulfilling the criteria demanded, including enlarged lymph nodes with lymphocyte depletion, presence of giant cells and a massive quantity of PCV2 detected by immunostaining [14].

In herd B signs resembling PMWS ("thin" and/or "hairy") were observed in two pigs, but no pig in this herd developed clinical PMWS. In herd C, no clinical signs PMWS were observed in any pig.

### Nucleotide sequence typing

A high similarity (99.7%) was found at the nucleotide level when comparing the full genome sequence of PCV2 obtained from the two Swedish herds (A and B), despite that they originated from a pig diagnosed with PMWS (herd A), and from a healthy pig (herd B). The similarity between these two Swedish sequences and that obtained from the Norwegian (herd C) was 95.5%. According to the proposed nomenclature for definition of PCV2 genotypes [18], the Norwegian isolate grouped into PCV2a whereas the two Swedish isolates grouped into PCV2b.

### PCV2 load in serum

**Figure 2:**
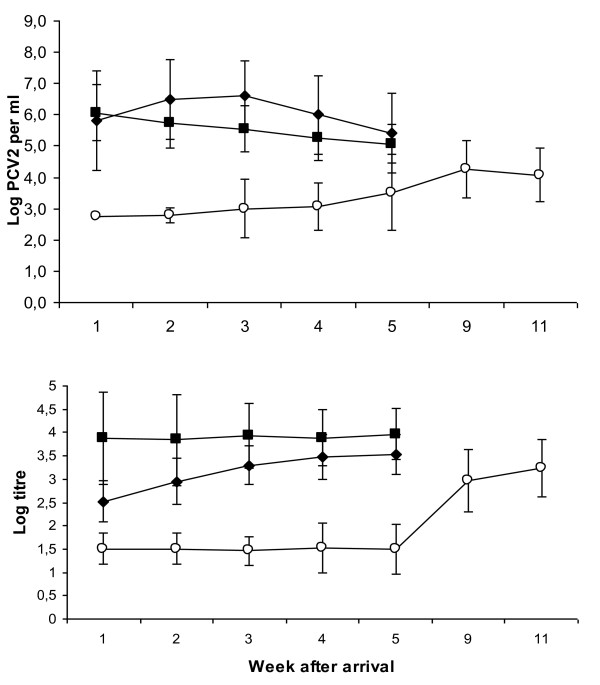
**Mean log levels of PCV2 DNA copy number per ml serum (upper) and log titre of antibodies to PCV2 (lower) in herds A (PMWS; filled diamonds), B (healthy, open squares) and C (healthy, open circles)**.

The PCV2 DNA copy number was determined by quantitative real-time PCR as an estimate of PCV2 viral load in serum (Figure 2). One week after arrival, the mean DNA copy number was similar (10^6 ^per ml serum) for pigs in herds A and B, but as seen in Table 3, pigs in herd A tended to express either high or low viral load (13 pigs above 10^7 ^DNA copies per ml serum and 7 pigs with less than 10^4 ^DNA copies per ml serum). The average viral load for pigs in herd A peaked at 10^6.5 ^per ml serum two weeks after arrival to the finishing unit, and then declined to 10^5.4 ^per ml in week five. In herd B, the average viral load decreased continuously from 10^6 ^per ml to 10^5 ^per ml serum in week 5. In contrast, no PCV2 DNA was detectable in serum of any pig in herd C during the first week after arrival. After five weeks in herd C the average viral load was 10^3.5 ^per ml serum, but values up to 10^6.4 ^per ml serum were recorded in individual pigs. During the extended period of sampling in herd C, the highest mean viral load (10^4.3 ^per ml serum) was recorded nine weeks after arrival. The highest incidence of pigs with a high viral load (exceeding 10^7 ^per ml serum) was found in the PMWS affected herd (A), predominantly during the early fattening period (Table 3). The load of PCV2 in the two pigs that developed PMWS increased to 10^10 ^per ml serum at the last occasion of sampling (day 18 and day 35, respectively).

**Table 3:** Number of pigs with a serum load of PCV2 exceeding 10^7 ^per ml serum.

Herd	Week 1	Week 2	Week 3	Week 4	Week 5
A	13/40	11/40	7/39	4/39	2/39
B	1/40	2/40	0/40	0/40	0/40
C	0/40	0/40	1/40	0/40	0/40

The animals were sampled during the first five weeks in two Swedish (Herds A and B) and one Norwegian (Herd C) finishing unit. Two of the forty pigs in herd A were diagnosed with PMWS, at 18 and 35 days after arrival, respectively. Based on fisher tests the number of pigs with virus load above 10^7^DNA copies per ml serum was significantly higher in herd A than in the other two herds in week 1, 2 and 3 (p < 0.05).

### Antibody titres to PCV2 in serum

In herd A, the mean antibody titre to PCV2 was 10^2.52 ^on arrival, and had increased to 10^3.52 ^five weeks later (Figure 2). Pigs in herd B had in general a higher level of antibodies to PCV2 (10^3.88^) than those in herd A when arriving at the finishing unit and remained at that level during the five weeks of sampling. Pigs in herd C were seronegative to PCV2 on arrival and remained negative during the first five weeks in the herd. By week nine these pigs had seroconverted to PCV2 and had a mean antibody titre of 10^3.24 ^at the last sampling occasion (11 weeks after arrival).

The two pigs in herd A that developed PMWS were both seronegative to PCV2 in the IPMA test (titre <10^1.6^) at the last occasion of sampling. In pig 6 that was still alive at the last sampling occasion, no indication of a serological antibody response to the increasing viral load was seen.

### Cortisol levels in saliva

Cortisol levels in saliva were lower in herd C (1.06 ± 0.14 ng per ml) than in herds A and B (1.80 ± 0.24 and 1.87 ± 0.19 ng per ml, respectively, p < 0.05). However, the cortisol levels were within the normal range in all herds.

## Discussion

In addition to different genotype of PCV2 in the Norwegian herd compared to the Swedish herds, the most remarkable differences between pigs from the three finishing herds were the levels and kinetics of their antibody response to PCV2, indicative of different starting situations at the time of allocation to the finishing herd. The highest levels of antibodies to PCV2 were recorded in serum from pigs in the healthy Swedish herd (B). In contrast, pigs in the healthy Norwegian herd (C) were seronegative to PCV2 at arrival and remained so during the first observation period of five weeks. The sampling period was therefore prolonged in this herd and a seroconversion to PCV2 took place between 5 and 9 weeks after arrival. Most pigs (29/40) in the Swedish PMWS-affected herd (A) were seropositive to PCV2 at arrival, but had lower titres than animals in herd B (p < 0.01). The antibody titres increased continuously in herd A, with exception for the two pigs that developed PMWS. These two pigs had initially low, declining antibody levels to PCV2 and were regarded as seronegative when displaying clinical symptoms of PMWS.

The observed serological responses to PCV2 are well in line with previous studies [12,17,19-21] supporting the relationship between PCV2 and PMWS also in finishing pigs. The lack of a proper antibody response in the two pigs that developed PMWS in herd A, further support earlier studies pointing out that neutralizing antibodies to PCV2 are protective against PMWS [22-25]. The IPMA-method used in this study does not discriminate between neutralizing and non-neutralizing antibodies, but a positive correlation between neutralizing antibodies and total amount of antibodies has previously been reported [22]. Indeed, the mean antibody titres to PCV2 increased steadily for the majority of pigs in the PMWS affected herd (A), indicating an ongoing infection with PCV2 on herd level.

The quantification of PCV2 DNA copies in serum revealed a similar viral load in pigs when entering the two Swedish finishing herds. A discrepancy was, however, that the mean serum viral load increased during the two first weeks for pigs that were allocated to the finishing unit affected by PMWS, whereas this load steadily decreased in serum samples collected from pigs in the healthy Swedish finishing herd. In clear contrast, no PCV2 DNA was detected in any serum sample collected during the first week in the Norwegian herd. Instead, low levels of PCV2 DNA could be detected in serum of a handful of these pigs after three weeks in the finishing unit, coinciding in time with seroconversion. Thus, most of these pigs were exposed to PCV2 at an age of 16 - 21 weeks, i.e. when pigs are regarded less likely to develop PMWS [2,9,10]. This discrepancy in age at the time of infection was also observed by Grau-Roma and others, as pigs in Spain were infected at a higher age than the Danish pigs [12]. Epidemiological studies of risk factors in PMWS dynamics have also shown that early infection increases the risk of PMWS [26-28].

It is notable that the viral load of PCV2 was higher in herd A than in the other herds, and that the number of pigs with serum viral levels above a proposed cut off at 107 per ml serum [15] as also supported by others [29] differed between the three herds. Herd A had a significantly higher number of pigs with serum PCV2 levels above 107 per ml during the first three weeks after arrival (p < 0.05), corresponding to the period of risk for PMWS in finishing herds [2,9,10]. This shows that although it is a crude tool, serum virus level may be used as an indicator of PMWS status on herd level, provided that the pigs are sampled at an appropriate time, i.e. during the first weeks in the finishing herd. It should however be noted that pigs with high viral load of PCV2 may mount a protective immune response to the infection, and do not necessarily develop PMWS [12,17]. In the present study, 18 of 20 pigs with a viral load above 107 per ml serum did not develop clinical PMWS or other PCV2 associated clinical signs.

Several external factors, including increased stress levels, have been suggested to contribute to the developments of PMWS as reviewed [13]. Social stress of pigs is associated with a negative effect on the antiviral immunity [30] and experimental studies have indicated that dexamethasone treatment can influence the pathogenic effect of PCV2, suggesting a role of stress and glucocorticoids in the PMWS aetiology [31]. Herd A distinguished from the two other herds by a higher mortality even during the periods free from PMWS. Furthermore, herd A became affected by PMWS after intensified routines with no empty time between some of the batches. Cortisol secretion was determined in order to test whether the more intensive management practices of herd A could have generated higher stress levels. The levels of cortisol determined in saliva collected from pigs in adjacent pens to those examined were similar in the two Swedish herds. Although these mean values were somewhat higher than those recorded for the Norwegian pigs, the cortisol levels for the three herds were all within the normal range [32] and no extreme stress-related behaviour such as tail-biting were recorded in any of the herds. Thus, long-term stress was unlikely to have caused the outbreak of PMWS in herd A.

Another factor that differed between the investigated herds was the predominating genotype of PCV2. Sequencing revealed that according to the nomenclature proposed by Segalés et al (2008), PCV2a was present in the Norwegian samples, whereas PCV2b was found in the two Swedish herds. In Sweden, PCV2b has been found in samples from herds diagnosed with PMWS as well as from healthy herds, whereas PCV2a has not yet been demonstrated in herds diagnosed with PMWS [33]. Currently there is a controversy regarding the possible influence of PCV2 genotype on the development of PMWS, and during experimental conditions PCV2a readily induces PMWS [34-36]. Furthermore, in a survey on the island of Ireland, both genotypes of PCV2 were demonstrated in a longitudinally study of a herd before and after it was affected by PMWS at farm level [37]. In Norway, sequencing of PCV2 from pigs in about 30 non-PMWS herds has revealed PVC2a in all herds. From February 2008, more than six months after terminating the sample collection of this trial, new cases of PMWS have been identified in Norway, and sequencing of PCV2 from pigs in these herds has demonstrated genotype PCV2b in all the 11 affected herds examined so far (ongoing project, unpublished data). This correlates well with the shift in predominant genotype from PCV2a to PCV2b observed during the PMWS epizooty in Switzerland [38].

Herd A was not officially deemed for PMWS on herd level until herd Z was the sole deliverer of growers, and herd Z itself was soon thereafter diagnosed with PMWS at a herd level. Nevertheless the historical data clearly indicate turbulence in herd A before the shift in source of growers. The problem occurred when herd A for the first time reduced the empty time between delivering slaughter pigs/introducing new finishing pigs to less than 24 hours ("instant repopulation") and the problem then accelerated as this error in management routine was repeated during the switch of piglet supplier. Interestingly, the growth of pigs that reached market weight was not affected by PMWS, but the herd suffered economically from an increased mortality and an increased incidence of underweighted pigs at slaughter.

Neither shedding of, nor seroconversion to PCV2, was seen during the first five weeks in the Norwegian finishing herd (C), and this comparatively late infection with PCV2 appears likely to contribute to why pigs in this herd were not affected by PMWS. Obviously, pigs originating from the Swedish sow pool that delivered animals to both herd A and B had a potential risk to develop PMWS. Yet, herd B remained free from PMWS, confirming the earlier observation that only occasional sow pool satellites will be affected by PMWS despite that the sows alter between the satellites [4]. The differences between affected and non-affected satellites have been linked to the intensity of the rearing strategies [8], and it is striking that logistics had forced herd A to exclude empty days between batches prior to the PMWS diagnosis and during the early course of the disease. The all in-all out concept was kept, but not the time for cleaning, disinfection or spontaneous microbial mortality. Furthermore, Herd A distinguished from herds B and C by a higher mortality even during the periods free from PMWS. The management practices in herd A might have been more intensive than in the two other herds and might have generated higher animal stress levels. Stress has been suggested, among other external factors, to contribute to the developments of PMWS [13]. Social stress of pigs is associated with a negative effect on the antiviral immunity [30] and experimental studies have indicated that dexamethasone treatment can influence the pathogenic effect of PCV2, suggesting a role of stress and glucocorticoids in the PMWS aetiology [31]. However, the levels of cortisol determined in saliva collected from pigs in adjacent pens to those examined were similar in the two Swedish herds. Although these mean values were somewhat higher than those recorded for the Norwegian pigs, the cortisol levels for the three herds were all within the normal range [32] and no extreme stress-related behaviour such as tail-biting were recorded in any of the herds. Thus, long-term stress was unlikely to have caused the outbreak of PMWS in herd A. Another thing that could be discussed in preventing PMWS is age at allocation. Pigs are still not mature when allocated to fattening enterprises, and the effect of one or two additional weeks before allocation may well be beneficial for prevention of PMWS, and indeed a correlation between immaturity of the immune system and PMWS has been suggested [39].

## Conclusions

In the present study, cortisol measurements excluded the presence of chronic stress in all herds. The most obvious difference between the two Swedish finishing herds and the Norwegian herd was the time of infection with PCV2 in relation to time of allocation, as well as the genotype of PCV2. The Swedish herds differed in PMWS status, and the herd that remained healthy had a higher serum antibody level to PCV2 when entering the finishing herd. It is also notable that the Swedish finishing herd that was affected by PMWS became so after errors in management routine, emphasising the important role of proper hygiene and general disease-preventing measures, whereas stress levels did not appear to play a major role. There was also a significant difference in the number of animals with viral titers above the cut-off at 10^7 ^copies/ml serum in the PMWS affected herd compared to the two other herds.

## Competing interests

The authors declare that they have no competing interests.

## Authors' contributions

IMB, CF, BL, GB, EM, ER, CMJ and PW initiated the study. They participated in its design and coordination and helped to draft the manuscript. Samplings and clinical evaluations were carried out by AL and BL in the Norwegian herd and by PW, CF, BG, EM and LES in the Swedish herds. GB performed the serological analysis, EM the cortisol analyses and PW the statistical analyses. IMB carried out the quantitative PCR, nucleotide sequencing, and sequence alignment, and drafted the manuscript. All authors read and approved the final manuscript.
